# Bridging soil resistome and clinical inference: A four-tier evidentiary framework

**DOI:** 10.1016/j.isci.2026.117815

**Published:** 2026-10-15

**Authors:** Simon Meski, Biljana Mojsoska, Karen A. Krogfelt, Carla Cruz-Paredes

**Affiliations:** 1 Department of Science and Environment, Roskilde University, 4000 Roskilde, Denmark; 2 PandemiX Center, Roskilde University, 4000 Roskilde, Denmark

**Keywords:** antimicrobial resistance, soil resistome, one health, clinical relevance, evidence tiers, horizontal gene transfer, environmental surveillance, risk assesment

## Abstract

Soils support a varied range of microbial communities and are important repositories of antimicrobial resistance genes. Sequence-based techniques, such as quantitative polymerase chain reaction and shotgun metagenomics, have substantially aided soil-resistome cataloging. Nevertheless, discovery alone does not indicate activity, transferability, exposure, or clinical relevance. This review contends that better clinical inference requires tying presence-based signals to function, mobility, and exposure routes, rather than just increasing survey volume. We offer a four-tiered evidentiary paradigm that distinguishes between detection, functional or expression-based evidence, mobile genetic element context, and exposure-linked evidence in environmental, animal, and human compartments. Because activity, mobility, and cross-compartmental similarity support several claims, layered, claim-aligned approaches are required for careful One Health interpretation.

## Graphical abstract

**Figure:**
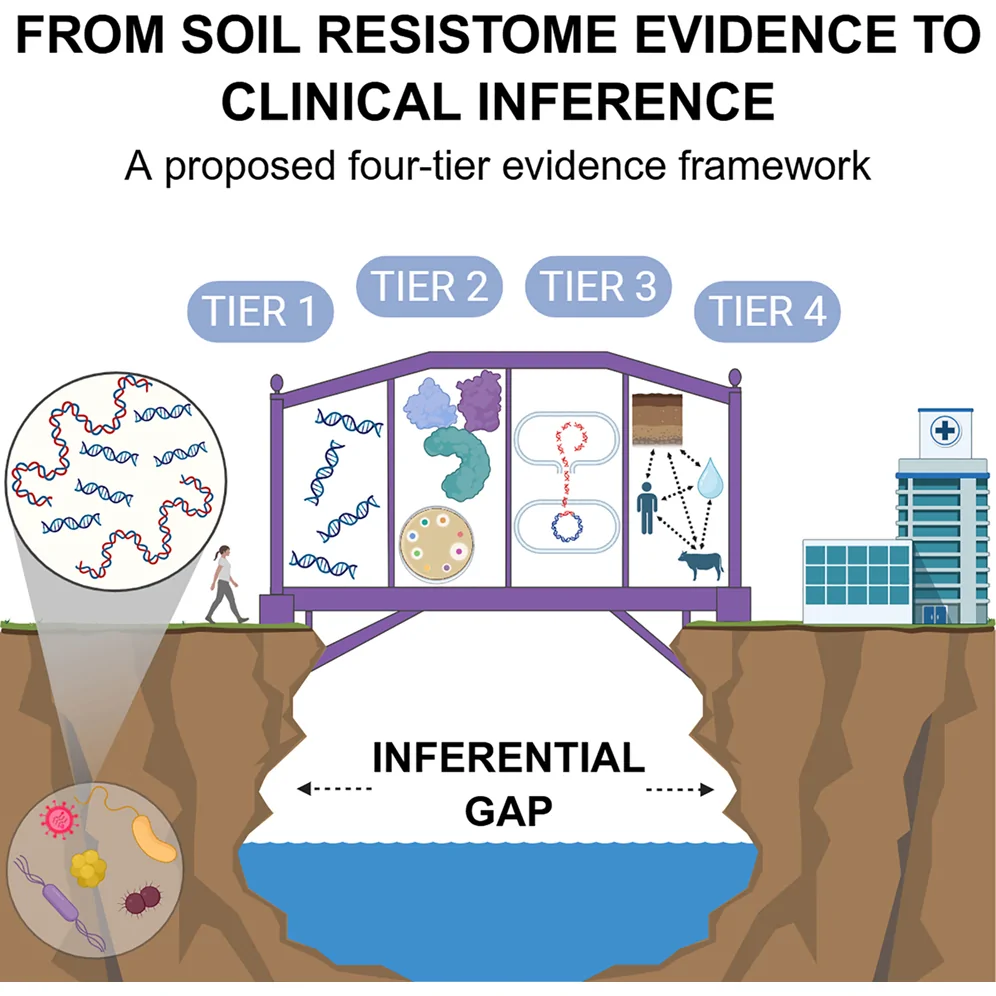

## Teaser

Soil science; Microbiology; Soil biology

## Glossary

### Antimicrobial resistance

The ability of microorganisms to survive antimicrobial exposure. In this review, antimicrobial resistance (AMR) is discussed as an environmental and clinical problem, however environmental detection alone is not treated as evidence of clinical burden.

### Antibiotic resistant bacteria (ARB)

A bacterium capable of growing at antibiotic concentrations that inhibit the growth of susceptible members of the same species. Resistance may result from intrinsic properties, resistance-conferring mutations, or acquired antimicrobial resistance genes (ARGs).

### Antibiotic resistance gene (ARG)

A gene or gene homolog associated with reduced antimicrobial susceptibility, such as β-lactamases, aminoglycoside-modifying enzymes, or efflux pumps. ARG detection indicates genetic potential, not necessarily expression, phenotype, mobility, or risk.

### Antibiotic resistance gene-mobile genetic element (ARG-MGE) linkage

Evidence that an ARG is physically associated with a mobile genetic element (MGE). This supports mobilization potential more strongly than ARG detection alone, especially when linkage is resolved by long reads, Hi-C, plasmid analysis, or junction-targeted assays.

### Cross-compartment tracking

Study designs that compare ARGs, mobile elements, plasmids, strains, or host bacteria across connected environments, such as soil, plants, animals, food, and humans. Similarity across compartments can suggest overlap but does not by itself prove directionality.

### ESKAPEE pathogens

A group of clinically important bacteria comprising *Enterococcus faecium*, *Staphylococcus aureus*, *Klebsiella pneumoniae*, *Acinetobacter baumannii*, *Pseudomonas aeruginosa*, *Enterobacter* spp., and *Escherichia coli*. These taxa are frequently associated with healthcare-associated infections and multidrug resistance.

### Functional metagenomics

An experimental approach in which DNA is cloned into a host organism and screened for a measurable phenotype, such as growth on antibiotic-containing media. It can identify resistance activity but may depend strongly on the host.

### Homology-based annotation

Assignment of gene identity or function by sequence similarity to reference databases. It is useful for screening resistomes, but similar sequences do not always encode the same phenotype or function in their native genetic context.

### Metagenomics

Sequencing-based analysis of genetic material recovered directly from a microbial community. In soil-resistome studies, it can identify ARG diversity and context, however DNA-level detection does not establish activity.

### Metaproteomics

Large-scale analysis of proteins recovered from a microbial community. It can support activity-related interpretation by showing protein-level evidence, although soil extraction, database coverage, and quantification remain challenging.

### Mobile genetic element (MGE)

A genetic unit capable of movement within or between genomes, including plasmids, integrons, transposons, and phages. MGE association can make ARG spread more plausible but does not guarantee transfer or persistence.

### One Health

A framework recognizing that human, animal, and environmental health are interconnected. In this review, One Health interpretation requires evidence connecting soil resistomes to plausible functional, mobile, and exposure-linked pathways.

### Quantitative polymerase chain reaction (qPCR)

A targeted molecular method used to quantify selected DNA sequences, including ARGs. It is sensitive and scalable but normally measures abundance rather than activity, mobility, or host compatibility.

### Resistome

The total collection of antimicrobial resistance determinants in a microbial community or environment. A soil resistome includes clinically relevant ARGs, ancient resistance homologs, intrinsic resistance systems, and genes with uncertain phenotype.

### Scopus

A bibliographic database used here to map publication patterns through keyword-based searches. In this manuscript, Scopus counts are treated as proxy indicators of evidentiary focus, not definitive classifications of individual studies.

## Review

### Introduction

Soil is one of the most microbially rich and physicochemically varied habitats on Earth and contains numerous antimicrobial resistance genes (ARGs) (see glossary) and antimicrobial resistance (AMR) determinants with potential significance to human health.^1^ Soil surveys often reveal ARG classes, such as tetracycline efflux pumps, β-lactamases, aminoglycoside-modifying enzymes, and multidrug efflux transporters.^2^ These are often identified using quantitative polymerase chain reaction (qPCR) arrays or shotgun metagenomics against reference databases.^1^^,^^2^

High-throughput sequencing has significantly expanded soil-resistome research and helped facilitate the creation of ARG reference libraries such as CARD and ResFinder/ResFinderFG, which catalog thousands of ARG sequences.^3^^,^^4^ However, metagenomic detection focuses on resistome potential rather than whether resistance determinants are active *in situ*, confer resistance under relevant circumstances, or are present in genetic settings that allow transfer to pathogens. Mechanistic interpretation is still restricted, in part because analytical chemistry procedures and functional omics methods such as metaproteomics and metabolomics are difficult to standardize across soil matrices.^5^^,^^6^ Furthermore, annotation algorithms often infer function from sequence homology to reference sequences with experimentally determined phenotypes, which might result in incorrect phenotype prediction. For example, Mahfouz et al.^7^ conducted a large-scale review of sequence-alignment-based resistance phenotype-prediction repositories and observed poor performance for genotype-to-phenotype prediction, particularly for efflux-related determinants. Such genotype-phenotype discrepancies can arise through paralog evolution or because annotated resistance-gene homologs are transcriptionally inactive or do not confer detectable resistance.

Additionally, soil-resistome composition is influenced by bacterial community composition and phylogeny because ARGs are carried and evolve within particular bacterial hosts rather than independently of their surrounding communities.^8^^,^^9^^,^^10^ Therefore, ARG detection should be interpreted alongside the identification and ecology of the host harboring it. An ARG carried by a soil lineage that rarely encounters human-associated bacteria may have fewer opportunities to reach clinical pathogens, whereas carriage by taxa that move across soil, wastewater, animal, or human-associated environments may provide more plausible routes of dissemination.^11^^,^^12^ Moreover, successful horizontal gene transfer and subsequent establishment generally become less likely with increasing phylogenetic distance because donor-recipient compatibility can constrain transfer, expression, replication, or persistence.^13^^,^^14^ However, this barrier is not absolute and may be overcome by broad-host-range MGEs or intermediate bacterial hosts.^15^ Host identity and ecology therefore give critical background for evaluating soil ARGs.

Studies that demonstrate presence, but not activity, mobility, or plausible exposure routes should be interpreted as characterizing ecological distribution rather than supporting claims of clinical significance. In this review, we argue that insufficient study volume is not the main barrier to clinical inference from soil-resistome data but rather insufficient evidence linking environmental detection to functional/activity-based evidence, mobility/mobile genetic element (MGE) contexts, and exposure-related evidence. We thus developed a four-tier evidentiary framework for analyzing soil-resistome data. The system builds up from presence-based detection (tier 1) to functional/activity-based evidence (tier 2), mobility/MGE context (tier 3), and exposure-linked evidence across connected compartments (tier 4). We further describe the assumptions and supporting data at each tier, with the goal of matching assertions regarding soil resistomes with the quality of the evidence given.

### To date environmental AMR evidence frameworks

Methods or frameworks for environmental resistome evidence confidence-scoring have previously been described, although they have generally addressed different analytical aims than those explored here.

Some frameworks on environmental resistomes have emphasized monitoring, regulatory relevance, critical control points, and risk assessment at the environmental AMR policy level, rather than as a claim-based interpretive hierarchy for individual soil research.^16^^,^^17^ Other studies have organized the field around causal pathway models such as the “Driver-Pressure-State-Exposure-Effect-Action” (DPSEEA) model, which are useful for mapping where evidence exists across the environmental-to-human pathway and is the most similar model of such discussed and proposed herein.^18^^,^^19^^,^^20^^,^^21^ These have highlighted that environmental AMR research is leaning toward upstream “pressure” and “state” evidence categories, with less direct evidence on human exposure and effect.^19^ Likewise, ARG-focused risk-ranking frameworks have been established to prioritize resistance genes based on criteria such as human-associated enrichment, mobility, and host pathogenicity, which has resulted in the identification of genes of higher public health significance.^22^ More recently, mobility-centered surveillance perspectives have argued that ARG mobility should be explicitly integrated into environmental risk assessment, with propositions on multi-tier surveillance designs.^17^

This review builds on these ideas of thought while addressing an interpretation issue: what sort of clinical conclusion is warranted by the information typically provided in soil-resistome studies? The evidentiary tiering framework described here is a soil-focused evidentiary framework designed to align claims and evidence by distinguishing between presence-based detection, functional/activity-based evidence, mobility/MGE context, and exposure-linked evidence. In this way, it should be understood as a synthesis tool for evaluating inferential strength.

### A four-tier evidentiary framework

To clarify how evidence from soil resistomes influences clinical inference, the proposed framework outlines the assertions supported by each layer. The tiers classify the evidence generated rather than the analytical method used, because a single method may contribute to more than one tier. Tier 1 supports assertions regarding ARG prevalence and distribution but not concerning resistance function or clinical origins. Tier 2 supports assertions about expression or resistance function under the conditions examined. Tier 3 supports claims regarding mobilization potential and, when transfer occurs, transferability under specific conditions. Tier 4 supports evidence for assertions of compartment connectivity and realistic exposure routes. Within tier 4, shared sequences imply compartment overlap, and statistical analyses measure their covariance. The detection of the same ARG in matching bacterial lineages and MGE settings across compartments, together with temporal and epidemiological linkages, gives greater evidence for transmission. Tier 4 evidence alone cannot prove transfer direction or demonstrate that an environmental reservoir contributed to a clinical illness. Either conclusion requires evidence integration across levels and analyses that exclude reverse transmission and shared sources.

Detecting ARG sequences in soil samples, including metagenomic reads, contigs, and MAGs (metagenome-assembled genomes), constitutes tier 1 evidence (Table 1). Depending on assembly quality and resolution, MAGs can associate chromosomally encoded ARGs with a bacterial host and genomic region, although plasmid-borne ARGs may require complementary long-read or other linkage approaches for reliable host assignment.^8^^,^^23^^,^^24^ Host association strengthens interpretation by relating the ARG to the ecology and phylogenetic position of its carrier. However, host assignment remains presence-based evidence unless accompanied by evidence of activity or mobility; physical linkage between an ARG and an MGE contributes to tier 3 evidence. The most common approaches within tier 1 are DNA-based surveys, usually based on qPCR arrays or shotgun sequencing.^1^^,^^2^ This type of information is presence based and are useful for ecological monitoring and comparisons across land use, management, or contamination gradients. However, tier 1 data do not prove biological activity and, alone, provide limited mechanistic inference since it do not demonstrate that a gene is transcribed, translated, phenotypically active, or related to an MGE context compatible with transfer. For example, van Goethem et al.^25^ detected 177 ARG homologs in Antarctic soils, the majority of which were efflux related, but no flanking mobility-associated sequences, which could suggest that these determinants were ancient chromosomal genes with other paralogous activity of low clinical significance. Likewise, high-throughput sequencing data are commonly annotated through alignment-based homology to determinants cataloged in repositories linked to human infection. Although this is a valuable first-time screening approach to describe ARG occurrence in soils, homology-based read alignment alone does not directly establish a resistance phenotype or a link to human-associated pathogens. Mahfouz et al.^7^ observed that genotype-to-phenotype prediction using AMR databases is still unsatisfactory, with overall balanced accuracies of 0.52 for CARD and 0.66 for ResFinder across 2,587 clinical isolates from five bacterial species. Their study also identified efflux-related determinants as the poorest identifiable, since they were prevalent in CARD outputs but had poor average specificity and were heavily influenced by regulatory contexts. Coincidentally, efflux-related genes are among the classes most frequently detected in soil metagenomics,^2^^,^^25^^,^^26^ thus, this reliance on homology-based annotation can distort phenotype-level interpretation when genomic data are considered in isolation. These considerations are essential for environmental or intrinsic resistance determinants. Detection of multidrug efflux systems, *bacA*-like genes, or rifampicin resistance-associated loci in a soil survey is tier 1 evidence and should be interpreted in light of background prevalence, allele-level specificity, native physiological function, bacterial host, and genomic context.^22^ *bacA*, for example, encodes an undecaprenyl-pyrophosphate phosphatase that is involved in cell-wall precursor recycling, while *rpoB*-mediated rifampicin resistance is determined by specific resistance-conferring mutations rather than the presence of *rpoB*.^27^^,^^28^ More generally, homologs belonging to the same ARG family may vary significantly in terms of ecological distribution, host range, mobility, and possible phenotypic features.^22^ Ubiquity does not render such determinants irrelevant; however, abundance alone does not establish clinical significance: tier 2 evidence is demonstrated activity or resistance function, tier 3 is documented mobilization, and tier 4 is tracking of the determinant or its bacterial carrier along a plausible exposure pathway. For non-mobile chromosomal determinants, the movement of the resistant bacterial lineage may be more important than horizontal transmission of the determinant. Despite these limitations, a query-defined proxy analysis of Scopus records suggests that presence-based detection studies constitute the largest tier within the mapped soil resistome literature (Figure 1).

**Table 1:** Tier framework

Evidentiary tier	What this tier includes	Examples of methods^a^ that can generate this evidence
Tier 1: presence based	presence-based detection of ARG occurrence, abundance, diversity, and taxonomic association or genomic context, without evidence of activity, mobility, or exposure.	qPCR, high-throughput qPCR, shotgun metagenomics, targeted capture, amplicon-based surveys, presence-focused ARG detection in metagenomic reads, contigs, or MAGs, single-cell genomics without functional validation.
Tier 2: functional/activity based	evidence that a determinant is expressed or functionally active under defined conditions, including transcriptomic, functional omics and phenotypic evidence.	functional metagenomics, susceptibility assays, heterologous expression, enzyme assays, (meta)transcriptomics, metaproteomics, (meta)proteomics, metabolomics, stable-isotope-informed activity assays.
Tier 3: mobility/MGE context	evidence linking ARG to mobility or MGE and therefore supporting inference about mobilization potential; transfer assays provide stronger transfer-related support within this tier but do not by themselves establish dissemination.	long-read metagenomics, plasmidomics, Hi-C linkage, integron/transposon analysis, phage-associated ARG analysis, conjugation assays, transformation assays, transduction studies, ddPCR-based ARG-MGE linkage, MAG-based or other assembly resolved analyses that establish physical ARG-MGE linkage.
Tier 4: exposure linked	evidence evaluated across ecologically relevant interfaces connected to humans or human-associated animals, including pathway-oriented tracking and comparative cross-compartment evidence	cross-compartment sampling designs, soil-plant and soil-animal tracking, strain-resolved genomics, plasmid tracking, cultivation-based tracing, quantitative transfer assays, exposure modeling.

a Methods are illustrative and are not exclusive to one tier; tier assignment depends on the evidence generated.

**Figure 1:**
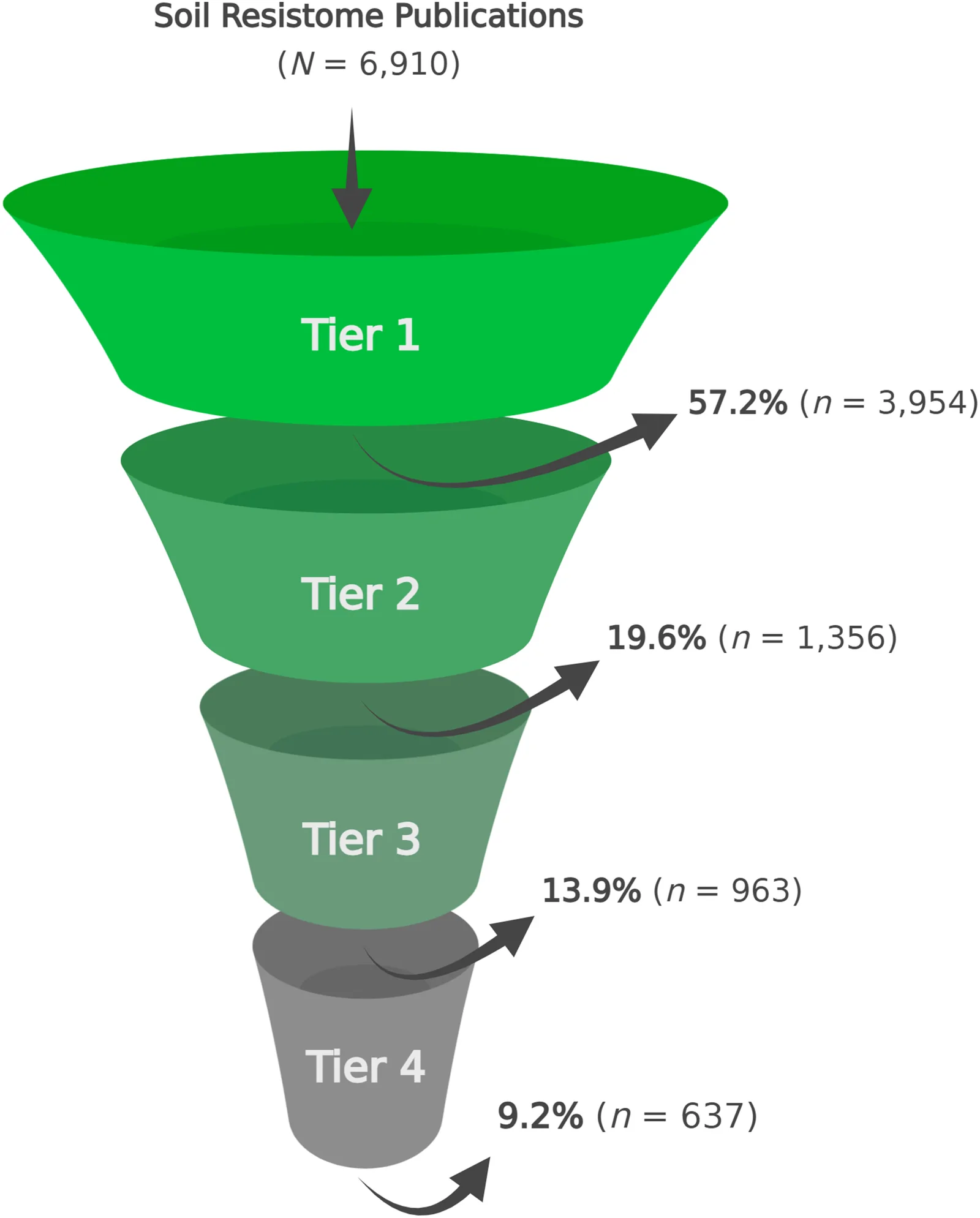
Evidence-tier distribution for soil resistome studies identified in Scopus (2005–2026) Evidence-tier distribution for soil resistome studies identified in Scopus (2005–2026). Publications were mapped to a four-tier framework (tier 1: presence-based detection; tier 2: function/activity evidence; tier 3: mobility/MGE context; tier 4: exposure-linked evidence) using keyword-based Scopus Advanced Search queries applied to a fixed base dataset (Q0; TITLE-ABS-KEY fields). Because a single publication could satisfy multiple tier queries, tier-exclusive counts were derived by hierarchical assignment to the highest tier matched (tier 4 > tier 3 > tier 2 > tier 1), with overlap quantified using combined queries (Q23, Q24, Q34, and Q234) and inclusion-exclusion correction for tier 2 exclusivity. Values shown are tier-exclusive counts and proportions of the base dataset (*N* = 6,910). Query strings are reported in the methods section and raw counts are reported in Table 2. Created in BioRender. Meski, S. (2026) https://BioRender.com/dgw3sdp.

Tier 2 represents functional/activity-based evidence. At this tier, evidence moves beyond DNA-level detection by showing that a resistance determinant is active under defined conditions, either through expression-related evidence or through experimental demonstration that it can confer a measurable resistance phenotype. In functional metagenomic screening, environmental DNA fragments are cloned into a surrogate host, commonly *Escherichia coli* (*E. coli*), and selected on via antibiotic-containing media to recover inserts that increase tolerance or resistance.^10^^,^^29^ In this setting, sequence-based prediction is linked to experimentally observed resistance-related activity under defined assay conditions. Multi-omics approaches can also contribute to tier 2 evidence by showing that DNA-level ARG signals are accompanied by transcriptional or functional activity. Metatranscriptomics can provide evidence of transcription under the sampled conditions, and metaproteomics or metabolomics may further support activity-related interpretation.^5^^,^^6^^,^^30^ Tier 2 evidence forms support for functional/activity-based evidence but depending on the analytical method chosen, data can be difficult to interchange for comparisons, since they have different levels of strength. A transcript is distinct from a resistance phenotype, and the detection of proteins or metabolites does not always indicate resistance *in vivo* and results are highly methodology dependent. Furthermore, even when a dataset containing ARGs is functionally validated, it may still require an appropriate mobility/MGE context (tier 3), such as association with conjugative plasmids, integrons, transposons, or phages, and suitable ecological conditions before transfer into a human-associated bacterial context becomes plausible. Also, successful transfer does not ensure establishment in recipient bacterial lineages of human interest (tier 4). Replicon compatibility, plasmid maintenance systems, regulatory context, codon usage, restriction-modification systems, CRISPR-Cas defenses, and fitness costs can all limit persistence in recipient lineages.^31^^,^^32^^,^^33^ Tier 2 evidence is therefore based on the host’s background. *E. coli* remains a valuable discovery host, but positive or negative results in a particular surrogate should not be generalized across bacterial lineages. When making broader ecological or clinical claims, a practical minimum would be cross-validation in one additional, strategically chosen host; for example, a representative of the likely environmental carrier, the native carrier if cultivable, or a clinically relevant recipient with a distinct physiological or phylogenetic background. Such cross-validation strengthens rather than universalizes the inference; hence, host selection should be based on the desired claim rather than a predetermined panel.^33^^,^^34^

Tier 3 represents mobility/MGE context. At minimum, tier 3 evidence shows physical genomic association between an ARG and an MGE, thereby supporting an inference of mobilization potential. Tier 3 is a higher-level category that looks at transferability rather than at functional activity or the presence of homologous ARG sequences. Mobilization potential is the ability of a resistance trait to spread across genomes and ecological compartments, driving population-level and clinical risk. Demonstrating physical linkage to MGEs or measurable transfer requires distinct methods and yields policy-relevant inferences about spread that neither tier 1 nor tier 2 evidence can provide. ARG-MGE linkage is more informative when the bacterial host and genomic location are known. A chromosomally encoded ARG without a known mobility context in an environmentally restricted taxon should be evaluated differently from the same determinant on a conjugative plasmid in a lineage that occurs across soil, wastewater, animal, or human-associated habitats. Tier 3 interpretation should therefore consider the identity and ecology of the carrier, the chromosomal or MGE-associated location of the ARG, and, where an MGE is involved, the element’s host range and compatibility with potential recipient lineages. Methods that can generate tier 3 evidence include bacterial plasmid capture,^35^ sequencing-based linkage methods,^36^^,^^37^ PCR identification of ARG-MGE junctions e.g., *intI1*, *tra*, *mob* genes,^35^^,^^38^ long-read sequencing, and mating studies which can provide stronger transfer-related support within this tier.^37^^,^^38^ Heuer et al.^35^ exogenously isolated 50 IncP-1 plasmids from manure-amended soils using an *E. coli* recipient; all carried class 1 integrons with *sul1* and additional resistance cassettes. This finding identified a plausible transfer-relevant context for soil-associated resistance determinants in bacterial recipient backgrounds of human interest. More broadly, detection of ARGs within integrons or transposons supports an inference of mobilization potential.^39^ However, tier 3 should not be interpreted as proof of dissemination at population or epidemiological scale. Even when broad-host-range plasmids are transferable in soil microcosms, transfer may remain infrequent, transient, or poorly maintained without sustained selection (≌ 10^–5^ to 10^–4^ transconjugants per donor or recipient^15^^,^^40^^,^^41^).^15^^,^^41^ In all, interpretation of dissemination beyond the experimental setting requires consideration not only of the presence of an MGE, but also of transfer frequency, host range, and post-transfer establishment. These, in turn, are constrained by replicon compatibility, plasmid maintenance functions, and recipient defense systems, including restriction-modification systems and CRISPR-Cas.^31^^,^^32^^,^^33^ Tier 3 therefore supports mobility-related inference, but in many cases, does not directly show dissemination or exposure-linked evidence.

Tier 4 represents exposure-linked evidence across connected compartments, including both pathway-oriented tracking and comparative cross-compartment evidence, although comparative overlap alone does not establish directional exposure. At this tier, ARGs are evaluated across connected compartments relevant to humans or animals, together with their bacterial hosts and MGE contexts where these can be resolved. Typical study designs therefore couple soil sampling with interfaces that could plausibly mediate transfer, such as soil-plant systems or soil-animal systems ideally with sufficient genetic resolution to distinguish shared background from directional movement. At tier 4, methodological rigor should stem from alignment between the model and the suggested exposure route, rather than from maximizing model complexity. A manageable minimum can focus on a single specified route and a claim-aligned bacterial host set; for example, the most probable environmental carrier and one clinically important receiver, with temporally ordered sampling of the source, key interface, and recipient compartment. Tracking a target ARG along with its carrier lineage or MGE and determining if it persists and imparts resistance in the recipient gives stacked evidence without the need for extensive host panels or multi-omics at each step. More complicated communities, additional recipients, or *in vivo* and epidemiological validation may then be reserved for candidates that persist or transfer in the initial model; nonetheless, the findings reflect route plausibility under the studied settings rather than clinical infection risk. Zhang et al.,^42^ for example, used a soil-snail-lettuce experimental setup to investigate movement along an exposure-relevant axis. And at a broader scale, Zhao et al.^11^ compared collections of soil metagenomes to human isolate *E. coli* genomes. They found that over the past few decades, soil ARG datasets and clinical resistance determinants have become more similar. They also found links between soil ARG-based risk metrics and population-level resistance rates in humans.^11^ Such analyses can support comparative inference about cross-compartment overlaps without by themselves establishing directional exposure or investigating in-detail molecular mechanisms. Cross-compartmental “sharing” should therefore be interpreted as a resolution-dependent description of sequence overlap rather than as evidence of transfer. The presence of the same ARG family, gene, or allele in environmental and clinical samples does not differentiate between ancient common ancestry, vertical inheritance, independent acquisition from a shared reservoir, movement of an ARG-carrying bacterial lineage, or horizontal transfer of an ARG-bearing MGE. Similarly, the compartment from which an ARG was originally isolated or recorded into a database reflects the source of the observation, not its evolutionary origin or route of spread. When the unit of movement is determined, the evidence becomes stronger: genome-wide similarity between ARG-carrying isolates supports bacterial lineage movement, whereas an identical ARG embedded within conserved flanking sequences and the same plasmid or other MGE background across different hosts supports genetic element movement. Environmental-to-human transmission necessitates temporal ordering of samples along a realistic exposure route, as well as epidemiological correlation when available. Even with such evidence, reverse transmission and common upstream sources must be considered; otherwise, the results should be classified as cross-compartment overlap or connection rather than transmission.

### Current evidentiary gap in soil-resistome research

ARG diversity has been widely cataloged on online repositories, but sequence detection alone does not establish resistance expression, mobilization, or transfer potential into human-associated environments.^7^^,^^17^^,^^30^ To investigate how the various tiers are represented in the soil resistome literature, we conducted a proxy-defined literature mapping in Scopus using a soil-resistome base query and additional keyword blocks corresponding to functional/activity-based evidence, mobility/MGE context, and exposure-linked evidence. Because publications may include many types of evidence, records matching higher-tier criteria were allocated hierarchically to the most appropriate tier, while the remaining base-corpus records were considered as tier 1 presence-based research. These numbers should therefore be taken as keyword-based indications of evidentiary focus rather than final classifications of each research.

In this mapped literature, presence-based detection appears to dominate (tier 1, 57.2%; *n* = 3,954/6,910), whereas functional/activity-based evidence, mobility/MGE context, and exposure-linked evidence show less often (Figure 1; Table 2). This tendency may represent methodological accessibility and the technological evolution of methods, but it also restricts the field’s capacity to go beyond description to mechanistic analysis. High-throughput sequencing and targeted molecular approaches are frequently employed due to their scalability, accessibility, and adaptability to a broad range of soil matrices. They enable wide comparative investigations, retrospective reanalysis, and connection with existing bioinformatic workflows. However, since homology-based annotation is still inadequate for phenotypic prediction, particularly for specific ARG classes, and does not prove functionality and mobilization, presence-based data may be overinterpreted when used as a proxy for clinical inference.^7^ This produces a recurring interpretive gap in which detection is treated as stronger evidence than it is.

**Table 2:** Scopus query counts and derived tier-exclusive counts

Quantity	Meaning	Value
Q0	base dataset	6,910
Q2	tier 2 query results	1,838
Q3	tier 3 query results	1,089
Q4	tier 4 query results	637
Q1	residual from Q0	3,346
Q23	tier 2 ∧ 3 query results	365
Q24	tier 2 ∧ 4 query results	159
Q34	tier 3 ∧ 4 query results	126
Q234	tier 2 ∧ 3 ∧ 4 query results	42
N_Tier4_	tier 4-exclusive results	637
N_Tier3_	tier 3-exclusive results	963
N_Tier2_	tier 2-exclusive results	1,356
N_Tier1_	tier 1-exclusive results	3,954

A lesser proportion of the literature addresses functional/activity-based evidence (tier 2, 19.6%; *n* = 1,356/6,910). Functional metagenomics and heterologous expression tests have found many factors that may produce resistance phenotypes under certain settings.^43^^,^^44^ These investigations show that certain environmental sequences may encode resistance-related activity under test conditions rather than passive sequence relics. However, functional confirmation is often achieved in model organisms under controlled settings, limiting ecological interpretation.^33^ A resistant phenotype shown in a model organism such as *E. coli* does not guarantee similar results in the environment, since expression and effects are influenced by genetic background, host physiology, and ecological selection.^33^ *In situ* expression can be inhibited by various promoters or regulatory networks; a gene that confers resistance in a lab host may impose fitness costs or be transcriptionally silent in its native host; and even if functional, the determinant may be incompatible with clinically relevant bacteria due to replicon incompatibility, codon usage, or host defense systems.

Even fewer studies examine the relationship between soil ARGs and MGEs (tier 3, 13.9%; *n* = 963/6,910), despite the importance of mobility for dissemination potential. Studies using plasmid capture, long-read sequencing, or integron/transposon analysis have shown that some soil-associated determinants occur in genetic contexts consistent with mobilization potential.^15^^,^^41^ Nonetheless, these studies are still less prevalent than detection-oriented surveys, and several recovered plasmids have a restricted host range, diminished stability, or poor transfer rates in microcosm experiments.

Exposure-linked evidence involving the movement of ARGs or ARG-carrying bacteria into human- or animal-relevant compartments are infrequent (tier 4, 9.2%; *n* = 637/6,910). There have been few investigations on soil-linked ARG signals in plant tissues, animal microbiomes, and food-production systems. Existing research supports the possibility of cross-compartment occurrence in certain circumstances, but it also highlights ecological filtration and genetic bottlenecks.^11^ Cross-compartment genomic comparisons reveal some overlap between soil resistomes and clinical isolates, but overlap does not imply directed exposure or transmission. Exposure-linked studies indicate that cross-compartment patterns are context-dependent and may represent distinct natural or anthropogenic situations.

This evidentiary imbalance has important consequences for interpretation. Over-reliance on presence-based data can encourage the assumption that detection implies clinical relevance, even though many soil ARGs may be ancient, non-expressed, non-mobile, or incorrectly assigned by homology-based methods. Limited functional/activity-based evidence and limited mobility/MGE-context studies also restrict the field’s ability to identify which determinants are most likely to be active, transferable, or compatible with human pathogens.^7^^,^^33^ Limited exposure-linked evidence further constrains interpretation of how soil-associated ARGs move into human-associated settings. The result is a literature base that is extensive in volume but uneven in interpretive depth. Methodological fragmentation widens this gap further. Studies focused on presence-based detection, functional/activity-based evidence, mobility/MGE context, or exposure-linked evidence are often conducted in isolation, making it difficult to reconstruct the ecological fate of specific ARGs or MGEs across tiers. A determinant that appears abundant in soil may never be tested for activity; a functionally validated determinant may never be examined for MGE linkage; and an MGE may never be followed across exposure-relevant compartments. Without cross-tier integration, the literature remains compartmentalized and less able to support claim-aligned interpretation.

Resource constraints may also contribute to the unequal distribution of evidence. Functional omics and cross-compartment designs require specialized infrastructure and more coordinated study design than DNA-based surveys alone.^5^^,^^6^ This may contribute to the continued dominance of presence-based detection and to slower standardization of multitier approaches across institutions. Even well-resourced studies may still produce datasets that are difficult to compare directly.

Finally, the evidential gap influences how soil resistance research is communicated to policymakers and the public. When detection-focused data predominate, detection might be confused with clinical significance, making environmental interpretation more challenging.^7^ The absence of higher-level data makes it difficult to establish whether soil ARGs should be considered clinically significant.

### Bridging environmental ARG detection and clinical inference

To integrate environmental detection with clinical inference, clear decisions must be made about which signals to study and which data combinations are sufficient to support a certain conclusion. Tier 1 surveys are useful for producing baseline maps, testing hypotheses, and tracking changes over time or across geography. They suggest potential candidates for further inquiry and give context for targeted sampling or action.^45^ However, presence-based detection should be understood as genetic potential rather than confirmation of phenotypic risk, and it should be utilized to select candidates rather than to infer clinical significance.^7^

Bridging detection of ARGs and clinical inference necessitates integrating evidence types that correspond to the strength of the claim being made. Functional validation approaches, such as functional metagenomics or heterologous expression, are required to provide functional/activity-based evidence and should be understood in conjunction with expression data. This combination may help interpret a locus as active under certain circumstances, but it does not demonstrate transferability or host compatibility.^33^ To enable mobility-related inference, physical connection to MGEs should be combined with transfer assays and host-range evaluation, if feasible. Linkage without transfer evidence or context creates doubt regarding permanence and spread.^35^^,^^46^^,^^47^ To facilitate better exposure-linked inference, cross-compartment tracing is often necessary, such as via matched environmental and host sampling or strain-resolved sequencing, to determine if environmental determinants are present in human-associated niches. However, attribution of clinical burden needs further evidence, such as epidemiological connection.^11^^,^^42^ Wastewater systems serve as an essential interface between clinical and soil resistomes because they receive human-associated antibiotic-resistant bacteria (ARBs), ARGs, and MGEs and produce effluent and biosolids, which may then enter environmental compartments. Hospital sewage may include large levels of human-associated bacteria and ARGs, but its contribution to municipal wastewater is dependent on the sewer-system environment.^48^ Wastewater treatment should not be assumed to be evenly eliminating or amplifying these signals: activated sludge may lower some ARGs while preserving other determinants and reconfiguring ARG-host and ARG-MGE connections.^36^ Major soil linkages occur via irrigation with treated or recovered wastewater-treatment plant effluent, as well as land application of sewage sludge or biosolids. Reclaimed-water irrigation may change soil ARG composition without increasing overall abundance over short periods,^49^ but biosolids amendment could increase ARG subtype richness and the relative abundance of ESKAPEE-associated species during crop cultivation.^50^ The fate of these inputs is determined by treatment performance, application history, residual selected agents, soil characteristics, competition with existing microbial communities, and the capacity of imported hosts or MGEs to persist and spread. Longitudinal, source-resolved sampling spanning hospital or municipal sewage, treatment stages, effluent or biosolids, and receiving soils is required to identify common background from likely movement of ARBs, ARGs, or MGEs and determine directionality.

To reduce overclaiming, the field should employ standardized descriptions for each evidentiary layer, such as “presence-based detection,” “functional/activity-based evidence,” “mobility/MGE context,” and “exposure-linked evidence.” A phased technique using wide monitoring to identify candidates, followed by specific function/activity, mobility, and exposure-linked investigations, may provide a more proportional path from environmental detection to stronger clinical inference.

Rapid advances in machine learning, especially deep learning, are broadening the potential use of artificial intelligence (AI) in every aspect of environmental AMR research. According to recent perspectives, AI is a computational layer that can be embedded across the workflows summarized in Table 1, from adaptive sampling and sequence analysis to ARG-host-MGE characterization, multi-omics integration, forecasting, anomaly detection, and decision support.^51^ Published applications demonstrate rather than exhaust this breadth. DRAMMA combines protein, genomic context, and evolutionary features to identify candidate ARGs lacking detectable homology^52^; machine learning identified 145 potentially mobile ARGs shared by chickens and farm environments^53^; and a 2026 study integrated 2,301 agricultural-soil metagenomes from 67 countries with soil, land use, and climate variables to model future ARG distributions.^54^ Such methods may filter datasets at sizes unreachable by manual analysis, prioritizing genes, hosts, mobile settings, or exposure routes for focused investigation. However, AI does not independently improve evidentiary strength. Tier assignment must be based on the underlying observations and their validation: a sequence-only prediction stays tier 1, but AI analyses on functional, transfer, or cross-compartment tracing data contribute only to the tier supported by those data. Reliability is also dependent on representative training data, uniform metadata, external validation, and uncertainty reporting, especially when clinically biased reference sets are applied to varied soil communities.^51^ Within these constraints, AI might help progress soil-resistome research beyond ever-expanding catalogs and toward coordinated prioritization and testing of the ARGs, hosts, and transmission channels most important to clinical emergence.

### Concluding remarks

We argue that the fundamental impediment to deriving clinically significant or therapeutically actionable conclusions from soil resistome data is not a lack of environmental surveys but a poor evidentiary translation from detection to inference. Presence-based, sequencing-derived archives are still required for baseline characterization and hypothesis formation. Within the proposed framework, novel or increasing determinants identified through tier 1 surveillance may serve as early-warning signals for prioritization but, when analyzed separately, they do not establish an emerging clinical threat. Soil may broadly be considered an ecological reservoir of resistance diversity; however, functional and mobility evidence is needed to strengthen the inference that a specific ARG has dissemination potential. Tier 4 evidence can position soil as a source, sink, or intermediate compartment within a plausible cross-compartment pathway. These interpretations are not mutually exclusive. However, concluding that a soil ARG contributed to the emergence of clinical resistance requires convergent evidence of its function, mobility, and movement along a plausible exposure pathway. Many soil-associated ARGs may be ancient, inactive, non-mobile, or ambiguously assigned by homology-based approaches, and relying too heavily on presence alone risks promoting the shortcut from discovery to putative clinical significance.

In conclusion, three parallel shifts drive practical progress. First, the study design must be claim-based, with methods chosen based on the minimum level of evidence needed to support the intended claim. Second, methodological integration across tiers is essential so that candidate genes or systems identified during surveillance may be intentionally escalated into focused functional tests, ARG-MGE linkage analysis, and cross-compartment or exposure-oriented sampling. Third, transparent reporting and shared standards are required, including explicit tier claims, unambiguous connecting metrics, uncertainty bounds, and direct identification of what each dataset does and does not give. By encouraging integrative, claim-driven research and clearer evidentiary criteria, the field can move beyond broad cataloging of soil genetic potential to a more focused set of mechanistically plausible, mobility-aware, and exposure-relevant targets for monitoring, risk interpretation, and policy.

## Methods

Publication counts were obtained from Scopus (Elsevier) on 17 March 2026 using the Advanced Search interface (https://www.scopus.com). All query strings and result counts are reported as used for the analysis. Scopus retrieval is based on bibliographic text fields (i.e., title, abstract, and author keywords) and so the resulting counts were treated as keyword-based proxies for evidentiary signals, rather than as definitive classification of each study’s full evidentiary depth.

A base corpus intended to capture the general soil resistome literature was defined as *a priori* as Q0 by combining soil-associated terms with antimicrobial-resistance/resistome terms and applying a publication-year restriction set at “from 2005”, as this is the approximate start of high-throughput sequencing experiments in microbiology:

TITLE-ABS-KEY (soil OR topsoil OR rhizosphere)

AND

TITLE-ABS-KEY (“antibiotic resistance” OR “antimicrobial resistance” OR resistome OR “antibiotic resistance gene∗” OR ARG)

AND

PUBYEAR >2004 AND PUBYEAR <2027

The resulting number of records N_Q0 from Q0 constituted the denominator for subsequent tier mapping.

Tier-specific retrieval was then performed by adding additional query blocks to Q0. Tier 1 was not retrieved with a separate Scopus query block. Instead, tier 1 was derived after retrieval of the higher-tier query blocks and subsequent overlap correction.

Tier 2 (functional/activity-based evidence) query block Q2: (added string):

TITLE-ABS-KEY (soil OR topsoil OR rhizosphere)

AND

TITLE-ABS-KEY (“antibiotic resistance” OR “antimicrobial resistance” OR resistome OR “antibiotic resistance gene∗” OR ARG)

AND

PUBYEAR >2004 AND PUBYEAR <2027

AND

TITLE-ABS-KEY (“functional metagenomic∗” OR heterologous OR “heterologous expression” OR “Escherichia coli” OR “susceptibility testing” OR “antibiotic susceptibility” OR MIC OR phenotype OR phenotypic OR “enzyme assay” OR metatranscriptom∗ OR metaproteom∗)

Tier 3 (mobility/MGE context) query block Q3 (added string):

TITLE-ABS-KEY (soil OR topsoil OR rhizosphere)

AND

TITLE-ABS-KEY (“antibiotic resistance” OR “antimicrobial resistance” OR resistome OR “antibiotic resistance gene∗” OR ARG)

AND

PUBYEAR >2004 AND PUBYEAR <2027

AND

TITLE-ABS-KEY (plasmid∗ OR plasmidome∗ OR integron∗ OR intI1 OR transpos∗ OR “integrative conjugative element∗” OR “integrative and conjugative element∗”)

Tier 4 (exposure-linked evidence) query block Q4 (added string):

TITLE-ABS-KEY (soil OR topsoil OR rhizosphere)

AND

TITLE-ABS-KEY (“antibiotic resistance” OR “antimicrobial resistance” OR resistome OR “antibiotic resistance gene∗” OR ARG)

AND

PUBYEAR >2004 AND PUBYEAR <2027

AND

TITLE-ABS-KEY (“wastewater irrigation” OR “wastewater reuse” OR “reclaimed water” OR “treated wastewater” OR “fresh produce” OR “ready-to-eat” OR edible OR “food chain” OR “farm to fork” OR endophyte∗ OR “human gut” OR gut OR fecal OR fecal OR livestock) AND TITLE-ABS-KEY (transfer OR transmission OR uptake OR internalization OR colonization OR “source tracking” OR “cross-compartment” OR continuum OR “soil-plant” OR “soil animal”)

A single publication could be captured by more than one tier query and so the raw N_Q*n* totals were not used as is. Instead, a hierarchical reassignment procedure was applied so that each record contributed to one tier only, using the order tier 4 > tier 3 > tier 2 > tier 1. To quantify overlap among higher-tier queries, combined queries were used to obtain Q23, Q24, Q34, and Q234. Tier-exclusive counts used in Figure 1 were then derived as follows:

Tier 4-exclusive = N_Q4

Tier 3-exclusive = N_Q3 - N_Q34

Tier 2-exclusive = N_Q2 – N_Q23 - N_Q24 + N_Q234

Tier 1-exclusive = Q0 - (Tier 2-exclusive + Tier 3-exclusive + Tier 4-exclusive)

## Acknowledgments

We are grateful to Associate Professor Emeritus Ole Skovgaard, Roskilde University, for fruitful discussions on the paper’s methodology, genomic arguments, and broader philosophy-of-science considerations. This work received no specific external funding. S.M.’s PhD fellowship is funded by the Department of Science and Environment, Roskilde University.

## Author contributions

Conceptualization, S.M, B.M., K.A.K., and C.C.P.; methodology, S.M.; formal analysis, S.M.; data curation, S.M.; visualization, S.M.; writing – original draft, S.M.; writing – review and editing, S.M., B.M., K.A.K., and C.C.P.; supervision, B.M., K.A.K., and C.C.P.; project administration, C.C.P. All authors reviewed and approved the final manuscript.

## Declaration of interests

The authors declare no competing interests.
